# S38. CHARACTERISING THE COGNITIVE CONSEQUENCES OF DISRUPTED BDNF-TRKB SIGNALLING AT PARVALBUMIN-EXPRESSING INTERNEURONS

**DOI:** 10.1093/schbul/sby018.825

**Published:** 2018-04-01

**Authors:** Adrienne Grech, Xin Du, Rachel Hill

**Affiliations:** Monash University

## Abstract

**Background:**

Schizophrenia is a debilitating syndrome characterised by three main symptom categories: positive, negative and cognitive. Cognitive symptoms emerge first, and currently do not have appropriate treatments, despite being a strong predictor of the severity and progress of the illness. Cognitive deficits are thought to be partly attributed to impaired synchronization of gamma frequency oscillatory activity. Gamma oscillations are generated by a subclass of GABAergic interneuron that expresses the calcium binding protein, parvalbumin (PV). PV-interneurons are supported by Brain Derived Neurotrophic Factor (BDNF) and recent evidence has found that cessation of BDNF support in PV- interneurons impairs gamma oscillations. All of these factors have been demonstrated to have a role in cognitive processing, but their dynamic relationship is not completely understood.

**Methods:**

The aims of this study were: 1) To generate transgenic mice where 50% of BDNF receptor (TrkB) gene is excised from PV-expressing neurons using the cre-lox recombination system and 2) To investigate the cognitive and behavioural consequences of disrupted BDNF signalling at inhibitory PV-expressing interneurons. Male and female mice underwent a battery of tests including: Y-Maze, Novel Object Recognition Task (NORT), Elevated Plus Maze, Locomotor and Cheeseboard Maze.

**Results:**

Sex-specific spatial memory impairments were found in PV-Cre x TrkB floxed mice with only males showing no preference for the novel arm in the Y-maze paradigm. Furthermore, male PV-Cre x TrkB floxed mice displayed a lack of cognitive flexibility in the cheeseboard maze for long term spatial memory. No significant differences were observed in measures of anxiety and activity, indicating that these were not confounding variables for cognitive measures.

**Discussion:**

This mouse line has not been cognitively characterised before and the results are of major interest. Subtle changes to cognition were observed and were sex-dependent. Interestingly, only males were observed to have changes in cognition, in line with human data. Human males with schizophrenia tend to exhibit more severe cognitive symptoms. Overall, the evidence from this study supports a role for BDNF-TrkB signalling at PV interneurons in regulating spatial memory performance. Future work will be investigating spatial search strategies of the Cheeseboard Maze, in order to elucidate further any cognitive differences between the genotypes. Additionally, future work will aim to specifically disrupt BDNF-TrkB signalling in the hippocampus and/or prefrontal cortex, as these two areas are highly implicated in both cognition and schizophrenia. It would also be of interest to use this genotype in a two-hit model, to further investigate the interaction of multiple factors and their impact on cognitive functions.

